# Extensive sharing of chloroplast haplotypes among East Asian Cerris oaks: The imprints of shared ancestral polymorphism and introgression

**DOI:** 10.1002/ece3.9142

**Published:** 2022-07-31

**Authors:** Yao Li, Lu Wang, Xingwang Zhang, Hongzhang Kang, Chunjiang Liu, Lingfeng Mao, Yanming Fang

**Affiliations:** ^1^ Co‐Innovation Center for Sustainable Forestry in Southern China, Key Laboratory of State Forestry and Grassland Administration on Subtropical Forest Biodiversity Conservation College of Biology and the Environment, Nanjing Forestry University Nanjing China; ^2^ School of Life Sciences Huaibei Normal University Huaibei China; ^3^ School of Agriculture and Biology Shanghai Jiao Tong University Shanghai China

**Keywords:** East Asian Cerris oaks, hybridization, introgression, phylogeography, *Quercus*, shared ancestral polymorphism

## Abstract

Shared ancestral polymorphism and introgression are two main causes of chloroplast DNA (cpDNA) haplotype sharing among closely related angiosperms. In this study, we explored the roles of these two processes in shaping the phylogeographic patterns of East Asian Cerris oaks by examining the geographic distributions of randomly and locally distributed shared haplotypes, which coincide with the expectations of shared ancestry and introgression, respectively. We sequenced 1340 bp of non‐coding cpDNA from *Quercus acutissima* (*n* = 418) and *Q. chenii* (*n* = 183) and compiled previously published sequence data of *Q. variabilis* (*n* = 439). The phylogenetic relationships among haplotypes were examined using a median‐joining network. The geographic patterns of interspecifically shared haplotypes were assessed to test whether nearby populations have a higher degree of interspecific cpDNA sharing than distant ones. We identified a total of 27 haplotypes that were grouped into three non‐species‐specific lineages with overlapping distributions. Ancestral haplotypes were extensively shared and randomly distributed across populations of the three species. Some young haplotypes were locally shared in mountainous areas that may have been shared refugia. The local exchange of cpDNA resulted in an excess of similar haplotypes between nearby populations. Our study demonstrated that the haplotype sharing pattern among East Asian Cerris oaks reflected the imprints of both shared ancestral polymorphism and introgression. This pattern was also associated with the relatively stable climates and complex landscapes in East Asia, which not only allowed the long‐term persistence of ancestral lineages but also connected the survived populations across refugia.

## Graphical abstract

We demonstrated that the haplotype sharing pattern among East Asian Cerris oaks reflected the imprints of both shared ancestral polymorphism and introgression. This pattern was also associated with the relatively stable climates and complex landscapes in East Asia, which not only allowed the long‐term persistence of ancestral lineages but also connected the survived populations across refugia.

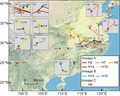

## 1 INTRODUCTION

Extensive sharing of chloroplast DNA (cpDNA) haplotypes is commonly observed among closely related angiosperms, which may be caused by both introgression and shared ancestral polymorphism (Acosta & Premoli, 2010; Heuertz et al., 2006; Nevill et al., 2014; Palmé et al., 2004; Petit, Csaikl, et al., 2002; Vitelli et al., 2017). On one hand, cpDNA is expected to be more frequently introgressed than nuclear DNA (Currat et al., 2008; Petit & Excoffier, 2009). This is because, in most angiosperms, maternally inherited cpDNA is only dispersed through seeds and thus has less potential for intraspecific gene flow (Petit et al., 2005); the low level of intraspecific gene flow hinders intraspecific homogenization. When two sister species come into contact, alien haplotypes from the invading species would not be diluted immediately by those of resident species (Currat et al., 2008; Du et al., 2011). Instead, they would be rapidly fixed in local populations under the enhanced effect of drift because haploid cpDNA has a much lower effective population size than diploid nuclear DNA (Currat et al., 2008; Herrera‐Arroyo et al., 2013; Palmé et al., 2003). These factors determine that chloroplast haplotypes are more likely to be shared among sister species through introgression (Acosta & Premoli, 2010). On the other hand, interspecific sharing of randomly distributed haplotypes may be a result of retained ancestral polymorphism (McGuire et al., 2007). According to the population genetics theory, population subdivision could increase a species' effective size, especially for cpDNA markers that experience low rates of migration (Hartl & Clark, 2007). This hints that ancestral lineages of cpDNA may be lost by drift less rapidly than nuclear DNA (Petit & Excoffier, 2009; Zhou et al., 2010). Furthermore, the low mutation rate of cpDNA may also decelerate lineage sorting and result in extensive sharing of ancestral haplotypes among sister species (Wolfe et al., 1987; Zhou et al., 2010). Given that cpDNA variation patterns more likely reflect the imprints of introgression (secondary contact) and shared geographic origin (ancestral sympatry) than speciation processes, it is essential to use information from as many closely related species as possible to track the evolutionary history of maternal lineages in angiosperms (Simeone et al., 2016).

*Quercus* (Fagaceae) is an ecologically important woody genus with more than 400 species spread throughout the Northern Hemisphere (Denk et al., 2017). Plastid phylogeny of oaks is in general decoupled from taxonomy, which may be caused by both shared ancestral polymorphism and ancient hybridization among ancestral populations (Simeone et al., 2016; Yang et al., 2021). For example, Ilex oaks (sect. *Ilex*) were resolved as non‐monophyletic using cpDNA data. Some Ilex oak species form the first diverging plastid lineage of the ‘Old World oak’ clade (subgenus *Cerris*), while the others are clustered with either Cerris oaks (sect. *Cerris*) or ring‐cupped oaks (sect. *Cyclobalanopsis*). A fourth lineage has been found in the westernmost populations of the Mediterranean *Q. ilex* (Simeone et al., 2016; Vitelli et al., 2017; Tekpinar et al., 2021; Yang et al., 2021; Zhou et al., 2022). A recent study has shown that shared ancestry alone is insufficient to explain the complex pattern; ancient hybridization also plays an important role (Zhou et al., 2022). Interspecific sharing of chloroplast haplotypes is more frequently observed among sympatric oaks within the same section (e.g., Belahbib et al., 2001; Cavender‐Bares et al., 2015; Dumolin‐Lapègue et al., 1999; Lyu et al., 2018; Petit et al., 1997; Petit, Csaikl, et al., 2002; Simeone et al., 2016; Pham et al., 2017; Whittemore & Schaal, 1991; Zhang, Hipp, & Gailing, 2015). This is particularly evident in European temperate white oaks (sect. *Quercus*), which share six lineages partitioned along a longitudinal gradient, reflecting a common history of long‐term isolation among separate refugia and massive introgression during the post‐glacial northward recolonization (Petit, Brewer, et al., 2002; Petit et al., 2004). However, the strong phylogeographic structure of European temperate white oaks is not found in eastern North American white oaks, suggesting a distinct biogeographic history in which past gene exchange occurred among populations from more diffuse refugia (Kremer & Hipp, 2020; Pham et al., 2017).

East Asia is home to a quarter of the world's oak tree species, but patterns of haplotype sharing between native oaks are largely unknown (Lyu et al., 2018; San Jose‐Maldia et al., 2017; Yang, Di, et al., 2016; Zeng et al., 2011). In this study, we explored the roles of shared ancestral polymorphism and introgression in shaping the haplotype sharing pattern among three closely related East Asian oaks, *Quercus acutissima*, *Q. variabilis*, and *Q. chenii*. Both nuclear and plastid data support that these species constitute a monophyletic group within sect. *Cerris* sister to the remaining members of the section in western Eurasia (Simeone et al., 2018; Hipp et al., 2020; Zhou et al., 2022). The divergence among the three species was estimated to have occurred during the early Oligocene to late Miocene (Hipp et al., 2020). Currently, *Q. acutissima* and *Q. variabilis* are among the dominant elements of East Asian temperate deciduous forests, while *Q. chenii* is restricted to deciduous broad‐leaved forests in eastern subtropical China (Huang et al., 1999). Morphological features including leaf and acorn size and distributions of leaf trichomes are used to distinguish them (Huang et al., 1999). Nevertheless, the occurrence of individuals with an intermediate phenotype suggests that putative hybrids may have occurred in their overlapping ranges (Liu, 1992; Hiroki & Kamiya, 2005).

Based on nuclear DNA data, recent studies have confirmed that the three closely related species are genetically coherent across their ranges despite introgressive hybridization (Chen, Zhang, et al., 2021; Fu et al., 2022; Li et al., 2022; Liang et al., 2022). The admixture pattern between *Q. acutissima* and *Q. chenii* was affected by past climate shifts. Most putative hybrids were concentrated in an ancient contact zone that may have existed during the mid‐Pliocene Warm Period but disappeared since the Early Pleistocene (Li et al., 2022). For *Q. acutissima* and *Q. variabilis*, strong signals of introgression were detected in sympatric populations throughout their ranges. Ecologically similar populations tended to share more introgressed regions of the oak genome (Fu et al., 2022). Compared with nuclear DNA, cpDNA is less effective in discriminating the three closely related species (Zhang et al., 2020). The extensive sharing of ancestral haplotypes resulted in an extremely low level of cpDNA differentiation between *Q. acutissima* and *Q. chenii* (Li et al., 2022). The wide distribution of these haplotypes also led to a non‐significant phylogeographic structure for each of the three species (Chen et al., 2012; Li et al., 2019; Zhang, Li, et al., 2015). More interestingly, a recent study has shown that some narrowly distributed haplotypes were private to sympatric populations where admixed individuals between *Q. acutissima* and *Q. chenii* are frequently observed, suggesting that introgression also plays an important role in shaping the haplotype sharing pattern at a local scale (Li et al., 2022). For these reasons, we infer that both shared ancestry and introgression may have left imprints on the phylogeographic patterns of East Asian Cerris oaks.

Here, we used two chloroplast intergenic spacers (*atpB*‐*rbcL* and *trnH*‐*psbA*) to examine the geographic patterns of chloroplast diversity in *Q. acutissima* and *Q. chenii*. The results were compared to cpDNA data previously obtained for *Q. variabilis* (Chen et al., 2012). Our two specific objectives were as follows: (1) to explore the roles of shared ancestral polymorphism and introgression in shaping the haplotype sharing patterns among the three species; (2) to assess the influence of East Asian climates and landscapes on the phylogeographic history of East Asian Cerris oaks.

## 2 MATERIAL AND METHODS

### 2.1 Sampling, DNA extraction, PCR amplification, and sequencing

**FIGURE 1:**
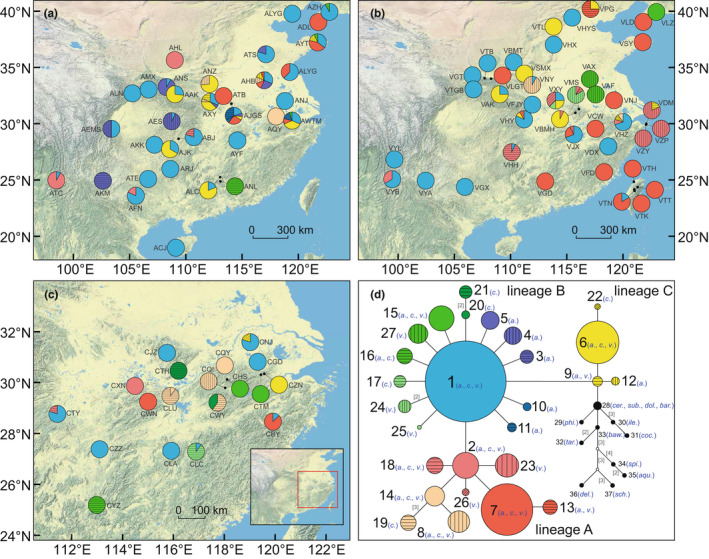
Geographic distribution and median‐joining network of the chloroplast DNA (cpDNA) haplotypes identified in this study. (a–c) Geographic distribution of the 27 cpDNA haplotypes detected in *Quercus acutissima* (a), *Q. variabilis* (b), and *Q. chenii* (c). Each pie chart represents a sampled oak tree population (see Table S1 for population codes) and each haplotype is represented with a different color/signature as shown in (d). The size of each section corresponds to the proportion of individuals with the specific haplotype in a given population. (d) Median‐joining network of the 37 cpDNA haplotypes detected in the three East Asian Cerris oaks (*a*., *Q. acutissima*; *c*., *Q. chenii*; *v*., *Q. variabilis*) and 13 outgroups (*cer*., *Q. cerris*; *sub*., *Q. suber*; *dol*., *Q. dolicholepis*; *bar*., *Q. baronii*; *phi*., *Q. phillyraeoides*; *ile*., *Q. ilex*; *coc*., *Q. coccifera*; *baw*., *Q. bawanglingensis*; *tar*., *Q. tarokoensis*; *spi*., *Q. spinosa*; *aqu*., *Q. aquifolioides*; *del*., *Q. delavayi*; *sch*., *Q. schottkyana*). The size of circles is proportional to the frequency of a haplotype across all populations. Open dots indicate inferred intermediate haplotypes not detected in this investigation. Numbers in brackets on branches indicate the number of mutations between haplotypes when branches represent more than one mutation.

Between 2014 and 2020, we sampled 33 populations of *Q. acutissima* (*n* = 418 individuals) and 19 populations of *Q. chenii* (*n* = 183 individuals), encompassing the majority of the distributions of both species in China (Figures 1 and S1, Table S1). Fresh and healthy leaves from 5 to 19 adult individuals spaced >50 m apart were collected at each sampling site and stored in silica gel. Total genomic DNA was extracted from 30 mg of dry leaf tissue of each individual using the Tiangen Plant Genomic DNA Kit (Tiangen, Beijing, China). Two chloroplast intergenic spacers, *atpB*‐*rbcL* and *trnH*‐*psbA* (Okaura et al., 2007), were amplified and sequenced for all the 601 samples following the methods described in Zhang, Li, et al. (2015).

We compiled previously published sequence data of the same two regions for 41 populations of *Q. variabilis* (*n* = 439 individuals) sampled throughout its entire distribution in China (Figures 1 and S1, Table S1; Chen et al., 2012). We also obtained sequence data from GenBank for 13 outgroups, including two western Eurasian Cerris oaks, *Q. cerris* and *Q. suber*; two Mediterranean Ilex oaks, *Q. coccifera* and *Q. ilex*; seven East Asian Ilex oaks, *Q. aquifolioides*, *Q. baronii*, *Q. bawanglingensis*, *Q. dolicholepis*, *Q. phillyraeoides*, *Q. spinosa*, and *Q. tarokoensis*; and two East Asian ring‐cupped oaks, *Q. delavayi* and *Q. schottkyana* (Table S2). It is necessary to include *Q. cerris*, *Q. suber*, *Q. coccifera*, and *Q. ilex* as outgroups because previous studies have shown that their haplotypes form the ‘Cerris‐Ilex’ lineage that is closely related to East Asian Cerris and Ilex oaks (Simeone et al., 2016, 2018). However, sequence data from the same individual were not available for the four species in GenBank. For this reason, we reconstructed haplotypes for them using sequences belonging to the same species but different individuals. For the *trnH*‐*psbA* region, we chose the most common haplotype of *Q. cerris* and *Q. suber* (Simeone et al., 2013, 2018) and the most common haplotype of the ‘Cerris‐Ilex’ lineage of *Q. coccifera* and *Q. ilex* (Simeone et al., 2016). We selected the ‘Cerris‐Ilex’ lineage for Mediterranean Ilex oaks because it is more closely related to Cerris oaks (Simeone et al., 2016). For the *atpB‐rbcL* region, we used the only available sequences in GenBank, which were collected from planted trees of the four species in the Botanical Garden of Zurich, Switzerland (Kamiya et al., 2003). For the other outgroups, we extracted *trnH*‐*psbA* and *atpB‐rbcL* sequences from corresponding complete chloroplast genomes reported previously (Li et al., 2021; Liu et al., 2019; Pang et al., 2019; Yang et al., 2017; Yang, Zhou, et al., 2016, 2018; Yang, Zhu, et al., 2018). Finally, a total of 1053 concatenated sequences were analyzed in this study, including five species of sect. *Cerris*, nine species of sect. *Ilex*, and two species of sect. *Cyclobalanopsis*. GenBank accession numbers of all the haplotypes detected in East Asian Cerris oaks and all the 13 outgroups were provided in Table S2.

### 2.2 Sequence variation and haplotype relationships

Sequences were proofread, aligned, and adjusted manually using BioEdit 7.2.5 (Hall, 1999). Insertions and deletions (indels) were treated as single mutation events and coded as substitutions (A/T) according to the simple gap coding method (Simmons & Ochoterena, 2000) as implemented in GapCoder (Young & Healy, 2003). An inversion of 32 bp detected in the *trnH*‐*psbA* region was replaced with its reverse complement and coded as a substitution, comparable with an indel (Xu et al., 2015). Length variations in mononucleotide repeats were excluded because of their tendency for homoplasy (Qiu et al., 2009). The resulting alignments were concatenated into a single matrix using FasParser 2.1.1 (Sun, 2017). Unique chloroplast DNA haplotypes were extracted by DnaSP 5.10 (Librado & Rozas, 2009). A median‐joining (MJ) network was constructed to visualize the relationships among haplotypes with PopART 1.7 (Bandelt et al., 1999; Leigh & Bryant, 2015).

### 2.3 Genetic diversity, differentiation, and demographic history

The number of haplotypes (*h*), haplotype diversity (*H*_d_), and nucleotide diversity (*π*) were calculated for each population using DnaSP 5.10. Average within‐population gene diversity (*h*_S_) and total gene diversity (*h*_T_) were computed for each species using Permut 2.0 (Pons & Petit, 1996). The presence of phylogeographic structure was assessed by testing the difference between genetic differentiation among populations (*G*_ST_) and the equivalent coefficient of differentiation considering similarities among haplotypes (*N*_ST_). A higher *N*_ST_ than *G*_ST_ usually indicates the existence of a phylogeographic structure, that is, closely related haplotypes occur more often in the same populations than less related ones (Pons & Petit, 1996). The significance of the difference between *G*_ST_ and *N*_ST_ was tested by a permutation test (*n* = 10,000) in Permut 2.0. To further investigate interspecific differentiation, a hierarchical analysis of molecular variance (AMOVA) was performed with Arlequin 3.5 (Excoffier & Lischer, 2010). This analysis partitions the total genetic variance into three levels: among species, among populations within species, and within populations. The significance of variance components and their associated fixation indices (*F*_CT_, *F*_SC_, and *F*_ST_) was assessed by 10,000 random permutations.

We performed Bayesian skyline plot (BSP) analyses to infer the past demographic dynamics of each species using the BDSKY package in BEAST 2.6.7 (Bouckaert et al., 2019; Drummond et al., 2005). The best‐fitting substitution model HKY was selected by ModelFinder (Kalyaanamoorthy et al., 2017). An uncorrelated lognormal relaxed clock and a Bayes‐skyline coalescent prior were used. The clock rate was set to 9.6 × 10^−10^ s/s/y (substitutions per site per year; Du et al., 2017). Two independent MCMC runs were performed for 1 × 10^9^ steps and sampled every 20,000 steps. The TRACER 1.7.1 (Rambaut et al., 2018) was used to assess convergence across runs.

### 2.4 Spatial autocorrelation

To investigate the spatial genetic structure at different geographic distance classes, we performed spatial autocorrelation analyses based on individual‐level geographic and haplotype genetic distance matrices. The first distance class was 0–50 km and the size of the following distance classes was increased in increments of 50 km. The significance of the autocorrelation coefficient (*r*) was tested for each distance class using a permutation test (*n* = 9999) that randomly shuffles all the individuals among sites. If the observed *r*‐value lies beyond the upper 95% bound of the null distribution of permuted *r* values, a positive spatial genetic structure is inferred. The significance of *r* was also assessed by bootstrap resampling (*n* = 9999) with replacement from the original dataset for a specific distance class. When the 95% confidence intervals (CIs) do not overlap zero, a significant spatial genetic structure is inferred. These analyses were conducted for each species pair and all three species using GenAlEx 6.5 (Peakall & Smouse, 2012).

### 2.5 Geographic pattern of interspecific cpDNA sharing

To test the hypothesis that nearby populations have a higher degree of interspecific cpDNA sharing than distant ones, we first calculated the gene identity (*J*), a measure of between‐population genetic similarity, for all pairs of populations belonging to different oak species. We then compared the distributions of *J* among three groups of population pairs: (1) population pairs separated by <300 km and sharing haplotypes (*J*_1_); (2) population pairs separated by <300 km, regardless of whether haplotypes were shared or not (*J*_2_); and (3) population pairs separated by ≥300 km (*J*_3_). We used 300 km as a threshold distance because we found that, for each species pair, the spatial autocorrelation coefficient declines smoothly when the size of the distance class exceeds 300 km, suggesting that the oak trees separated by <300 km were more genetically similar to each other. The measures of interspecific gene identities and their means (*M*_1_, *M*_2_, and *M*_3_ corresponding to *J*_1_, *J*_2_, and *J*_3_) were computed according to Dumolin‐Lapègue et al. (1999) and Belahbib et al. (2001). The distributions of *J* were compared statistically using a Wilcoxon rank‐sum test. All the analyses were performed for each species pair and all three species using R 3.5.1 (R Core Team, 2018).

## 3 RESULTS

### 3.1 Sequence variation and haplotype relationships

The lengths of consensus sequences after alignment of *atpB*‐*rbcL*, *trnH*‐*psbA*, and concatenated cpDNA were 726, 614, and 1340 bp, respectively. Seventeen substitutions and three indels (5–8 bp in length) were detected in the *atpB*‐*rbcL* region (Table S3). Seventeen substitutions, 12 indels (1–24 bp in length), and one 32‐bp inversion were detected in the *trnH*‐*psbA* region (Table S3). A total of 37 haplotypes were identified based on these polymorphisms, of which 27 haplotypes (H1–H27) were specific to East Asian Cerris oaks, while the remaining 10 (H28–H37) only occurred in outgroups (Figure 1d).

**FIGURE 2:**
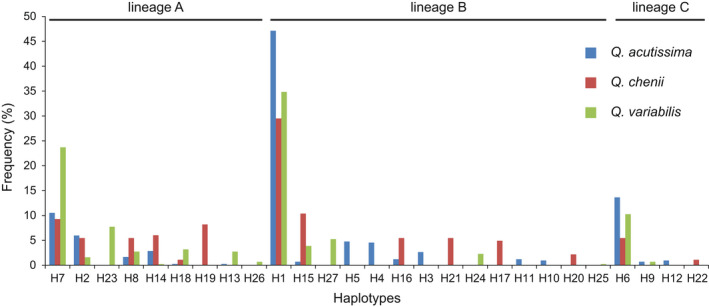
Frequency of chloroplast haplotypes in each of the three East Asian Cerris oak species, *Quercus acutissima*, *Q. chenii*, and *Q. variabilis* (Table S4). Within each lineage, haplotypes are sorted in a decreasing order based on the frequency across the three species.

Among the three East Asian Cerris oak species, *Q. acutissima* had the highest number of haplotypes (17), followed by *Q. variabilis* (15) and *Q. chenii* (14). Eight haplotypes (H1, H2, H6–H8, H14, H15, and H18) were shared by all three species, three (H9, H13, and H16) were shared by two species, and the remaining 16 (H3–H5, H10–H12, H17, and H19–H27) were private to a single species (Figure 1d; Table S4). Although the interspecifically shared haplotypes make up for less than half (40.7%) of the total number of distinct haplotypes, they were found to occur in 83.3% of the investigated individuals, including 84.9% of *Q. acutissima* individuals, 78.1% of *Q. chenii* individuals, and 83.8% of *Q. variabilis* individuals (Table S4). The most common haplotypes were H1, H7, and H6, found in 38.8%, 15.9%, and 10.8% of the sampled individuals, respectively (Figure 2 and Table S4). The other eight haplotypes shared by at least two species were represented by 0.6–4.0% (mean 2.2%) of the individuals. The 16 haplotypes unique to a single species were detected in 0.1–3.3% (mean 1.0%) of the individuals (Table S4).

The median‐joining network grouped the 27 haplotypes of East Asian Cerris oaks into three non‐species‐specific lineages (Figure 1d). Among those, lineages A and B were consistently separated by a transition (A/G) in the *atpB*‐*rbcL* region; lineages B and C differed by an 8‐bp indel in the *trnH*‐*psbA* region (Figure 1d; Table S3). Haplotypes shared among the three species were often detected at a relatively high frequency and the internal parts of the network, such as H2 (4.0% of all the sampled individuals) and H7 (15.9%) in the lineage A, H1 (38.8%) in the lineage B, and H6 (10.8%) in the lineage C (Figure 1d; Table S4). In contrast, haplotypes private to a single species were always found to be at a relatively low frequency and the tips of the network, such as H19 (1.4%) and H26 (0.3%) in the lineage A, H10 (0.4%) and H25 (0.1%) in the lineage B, and H12 (0.4%) and H22 (0.2%) in the lineage C (Figure 1d; Table S4). The lineage comprising all the 13 outgroups was separated by the lineage C by another 8‐bp indel in the *trnH*‐*psbA* region (Figure 1d; Table S3). Among the outgroups, two East Asian Ilex oaks (*Q. dolicholepis* and *Q. baronii*) and two reconstructed members of western Eurasian Cerris oaks (*Q. cerris* and *Q. suber*) shared the haplotype closest to the lineage C (i.e., H28). The other nine outgroups were separated from H28 by one to three mutational steps in the *trnH*‐*psbA* (*Q. bawanglingensis*) or *atpB*‐*rbcL* region (*Q. phillyraeoides* and *Q. ilex*), or by three to 10 mutational steps in both regions (the other six outgroups).

The three lineages of East Asian Cerris oaks presented an overlapping distribution (Figures 1 and S2–S4). The lineage A included an interior haplotype (H2) geographically scattered in southwestern, central, and eastern China, with eight satellite haplotypes mainly distributed in central and eastern China. The lineage B displayed a star‐like pattern, with a central (median) haplotype (H1) widely detected across the entire distributions of the three species, and 13 derived haplotypes occurring in one to six populations in southwestern, central, and eastern China. The lineage C was composed of four haplotypes. Among those, H6 was found in 15 populations of central and eastern China, whereas the other three were confined to one to three populations in eastern China (Figures 1 and S5–S31).

### 3.2 Genetic diversity, differentiation, and demographic history

The number of haplotypes (*h*), haplotype diversity (*H*_d_), and nucleotide diversity (*π*) in each population ranged from 1 to 6 (mean 1.65), zero to 0.842 (mean 0.163), and zero to 0.00263 (mean 0.00032), respectively (Table S1). There were neither significant linear nor quadratic associations of genetic diversity with latitude and longitude (all *p*‐values >.05). Among the three East Asian Cerris oaks, the total gene diversity (*h*_T_) was found to be three to nine times greater than the average within‐population gene diversity (*h*_S_). The highest value of *h*_T_ was observed in *Q. chenii* (0.902), followed by *Q. variabilis* (0.793) and *Q. acutissima* (0.747). The largest value of *h*_S_ was detected in *Q. acutissima* (0.252), followed by *Q. variabilis* (0.120) and *Q. chenii* (0.100) (Table 1).

**TABLE 1:** Genetic diversity and differentiation of populations of *Quercus acutissima*, *Q. chenii*, and *Q. variabilis*.

Species	*h*_T_ (SE)	*h*_S_ (SE)	*G*_ST_ (SE)	*N*_ST_ (SE)
*Q. acutissima*	0.747 (0.060)	0.252 (0.051)	0.663 (0.068)	0.638 (0.076)
*Q. chenii*	0.902 (0.048)	0.100 (0.038)	0.889 (0.043)	0.870 (0.064)
*Q. variabilis*	0.793 (0.039)	0.120 (0.032)	0.849 (0.040)	0.846 (0.042)
Three species	0.805 (0.030)	0.163 (0.025)	0.798 (0.031)	0.799 (0.034)

Abbreviations: *h*_T_, total gene diversity; *h*_S_, average within‐population gene diversity; *G*_ST_, genetic differentiation among populations; *N*_ST_, genetic differentiation among populations taking similarities between haplotypes into account; SE, standard error.

For each species, genetic differentiation among populations was substantial as indicated by the high values of *G*_ST_ and *N*_ST_ (Table 1). However, comparisons of these two measures did not reveal any significant phylogeographic structure. *N*_ST_ was not significantly larger than *G*_ST_ in any of the three species (*Q. acutissima*: *G*_ST_ = 0.663, *N*_ST_ = 0.638, *p* = .885; *Q. chenii*: *G*_ST_ = 0.889, *N*_ST_ = 0.870, *p* = .817; *Q. variabilis*: *G*_ST_ = 0.849, *N*_ST_ = 0.846, *p* = .568). *N*_ST_ and *G*_ST_ were not significantly different when the three datasets were combined (*G*_ST_ = 0.798, *N*_ST_ = 0.799, *p* = .438). The results of AMOVA indicated a relatively low level of cpDNA differentiation between each species pair (*F*_CT_ = 0.029–0.031, *p* = .039–.076) as well as among the three species (*F*_CT_ = 0.029, *p* = .021) (Table 2). Most of the total genetic variation (68.79–82.45%) was partitioned among populations within species (Table 2). The BSP showed that the three species maintained a relatively stable population size since the Middle Pleistocene (Figure S32).

**TABLE 2:** Analyses of molecular variance (AMOVAs) for populations of *Quercus acutissima*, *Q. chenii*, and *Q. variabilis*

Source of variation	*df*	*SS*	*VC*	*PV* (%)	Fixation index	*p*
*Three species*						
Among species	2	35.12	0.03	2.88	*F*_CT_ = 0.029*	.021
Among populations within species	90	745.76	0.72	74.40		
Within populations	947	208.84	0.22	22.72		
*Q. acutissima* and *Q. chenii*						
Among species	1	14.89	0.03	3.13	*F*_CT_ = 0.031	.050
Among populations within species	50	390.13	0.65	68.79		
Within populations	549	146.10	0.27	28.08		
*Q. acutissima and Q. variabilis*						
Among species	1	19.31	0.03	2.90	*F*_CT_ = 0.029*	.039
Among populations within species	72	547.18	0.64	71.25		
Within populations	783	181.09	0.23	25.85		
*Q. chenii* and *Q. variabilis*						
Among species	1	17.74	0.03	2.91	*F*_CT_ = 0.029	.076
Among populations within species	58	554.23	0.91	82.45		
Within populations	562	90.48	0.16	14.64		
*Q. acutissima*						
Among populations	32	191.54	0.45	59.36	*F*_ST_ = 0.594**	<.00001
Within populations	385	118.35	0.31	40.64		
*Q. chenii*						
Among populations	18	198.59	1.13	86.96	*F*_ST_ = 0.870**	<.00001
Within populations	164	27.75	0.17	13.04		
*Q. variabilis*						
Among populations	40	355.64	0.82	83.83	*F*_ST_ = 0.838**	<.00001
Within populations	398	62.73	0.16	16.17		

Abbreviations: *df*, degree of freedom; *SS*, sum of squares; *VC*, variance components; *PV*, percentage of variation. *p < .05; **p < .01.

### 3.3 Spatial autocorrelation

**FIGURE 3:**
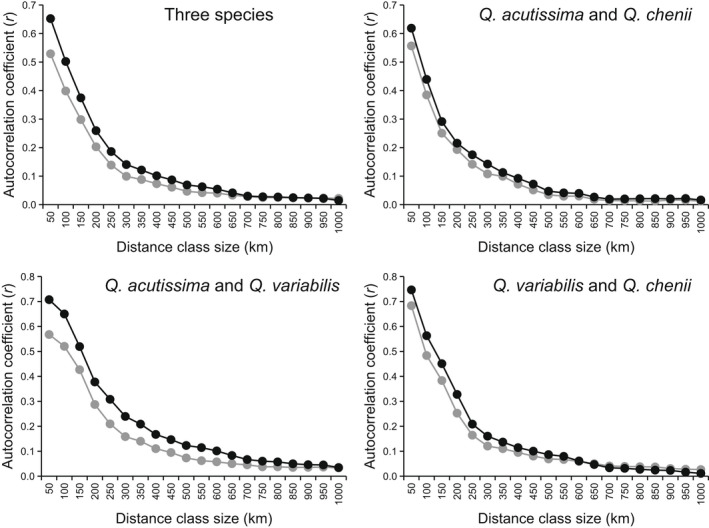
Spatial autocorrelation analyses based on individual‐level geographic and haplotype genetic distance matrices. The first distance class was 0–50 km and the size of the following distance classes was increased in increments of 50 km. Gray and black lines correspond to the results for datasets including and excluding the individuals with the most widespread haplotype H1, respectively.

Spatial autocorrelation analyses indicated that for the three species and each species pair, a significant and positive genetic structure occurred at all the 20 distance classes when the size of the distance class was increased in increments of 50 km (all *p*‐values <.01). The highest value of the spatial autocorrelation coefficient (*r*) was observed for the distance class of 0–50 km (0.529 ≤ *r* ≤ 0.683). The *r* values decreased with the increasing size of the distance class and began to decline smoothly when the size of the distance class exceeds 300 km (Figure 3). A consistent trend was observed for the datasets excluding the individuals with the most widespread haplotype H1, while the corresponding *r* values of each distance class were slightly higher than those of the original datasets (Figure 3).

### 3.4 Geographic pattern of interspecific cpDNA sharing

We compared interspecific gene identities among three groups of population pairs to test whether nearby populations have a higher level of interspecific cpDNA sharing than distant ones. When the most widespread haplotype H1 was considered, there was no significant difference between the mean of interspecific gene identities for population pairs separated by <300 km (*M*_2_) and that for population pairs separated by ≥300 km (*M*_3_) (*P*_23_ ≥ 0.05 for all species pairs; Table 3). However, when H1 was excluded, *M*_2_ was found to be 1.9–3.3 times greater than *M*_3_ for the ‘*Q. acutissima*‐*Q. variabilis*’ and ‘*Q. acutissima*‐*Q. chenii*’ pairs (*P*_23_ ≤ 0.002), but such a difference was not significant for the ‘*Q. chenii*‐*Q. variabilis*’ pair (*P*_23_ = 0.920) (Table 3). Additionally, the mean of interspecific gene identities for population pairs separated by <300 km and sharing haplotypes (*M*_1_) was found to be always significantly larger than *M*_2_ and *M*_3_ for all species pairs (*P*_12_ and *P*_13_ ≤ 0.004), regardless of whether H1 were considered or not (Table 3).

**TABLE 3:** Comparison of interspecific gene identities among three groups of population pairs

Species group	Population pairs separated by <300 km and sharing haplotypes		Population pairs separated by <300 km		Population pairs separated by ≥300 km		*P*_12_	*P*_13_	*P*_23_
	*N*	*M*_1_	*N*	*M*_2_	*N*	*M*_3_			
Including haplotype H1									
*Q. acutissima* and *Q. variabilis*	55	0.378	100	0.208	1253	0.215	0.000	0.000	0.408
*Q. acutissima* and *Q. chenii*	19	0.462	45	0.195	582	0.171	0.000	0.000	0.496
*Q. chenii* and *Q. variabilis*	19	0.400	98	0.078	681	0.157	0.000	0.000	0.069
All three species	93	0.400	243	0.153	2516	0.189	0.000	0.000	0.406
Excluding haplotype H1									
*Q. acutissima* and *Q. variabilis*	23	0.548	60	0.210	660	0.110	0.000	0.000	0.002
*Q. acutissima* and *Q. chenii*	9	0.475	24	0.178	336	0.054	0.004	0.000	0.000
*Q. chenii* and *Q. variabilis*	7	0.605	72	0.059	378	0.083	0.000	0.000	0.920
All three species	39	0.541	156	0.135	1374	0.089	0.000	0.000	0.002

*Note*: *N*, number of pairs of populations; *M*_1_, *M*_2_, and *M*_3_, means of interspecific gene identities for population pairs separated by <300 km and sharing haplotypes (*J*_1_), for population pairs separated by <300 km (*J*_2_), and for population pairs separated by ≥300 km (*J*_3_); *P*_*ij*_, *p*‐values for comparison between the distributions of *J*_*i*_ and *J*_*j*_ using Wilcoxon rank‐sum test.

**FIGURE 4:**
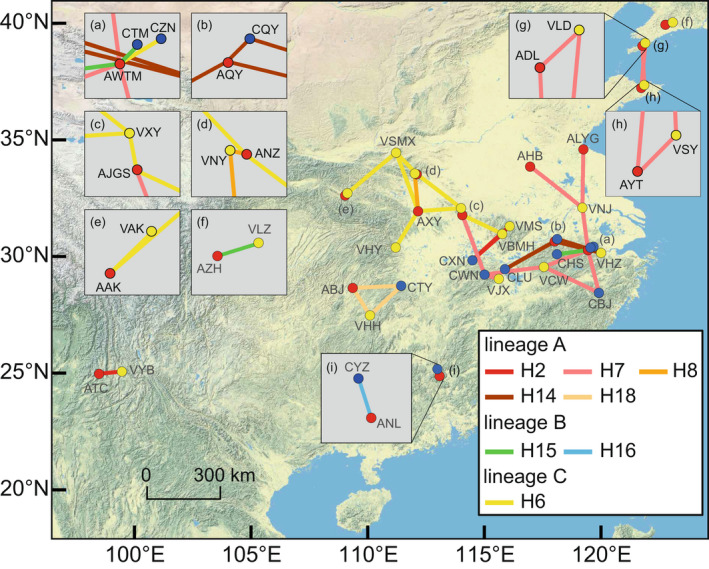
Geographic distribution of nearby populations (separated by <300 km) belonging to different oak species and sharing chloroplast DNA (cpDNA) haplotypes except for H1. Red, blue, and yellow points represent populations of *Quercus acutissima*, *Q. chenii*, and *Q. variabilis*, respectively (see Table S1 for population codes). The colors of line segments represent the haplotypes shared by the populations at two endpoints.

We found 39 pairs of nearby populations (separated by <300 km) interspecifically sharing eight cpDNA haplotypes except for H1 (Figure 4; Table S5). Among those, nine were ‘*Q. acutissima*‐*Q. chenii*’ pairs, 23 were ‘*Q. acutissima*‐*Q. variabilis*’ pairs, and seven were ‘*Q. chenii*‐*Q. variabilis*’ pairs. The interspecific gene identity was not significantly different among species pairs (mean ± SD, *Q. acutissima*‐*Q. chenii*: 0.475 ± 0.420, *Q. acutissima*‐*Q. variabilis*: 0.548 ± 0.386, *Q. chenii*‐*Q. variabilis*: 0.605 ± 0.432; all *p*‐values >.05; Table 3).

Thirteen of the 39 population pairs shared five haplotypes with a narrow distribution (i.e., H8, H14, H15, H16, and H18; Figure 4; Table S5). Specifically, lineage A H8 was shared by *Q. acutissima* and *Q. variabilis* populations in the Funiu Mountains, central China (Henan and Hubei Provinces); its direct ancestor H14, derived from H2, was restricted to populations of the three species in the Tianmu, Jiuhua, and Lu Mountains, southeastern China (Jiangxi, Anhui, and Zhejiang Provinces). Lineage A H18, a less frequent satellite of H2, was mainly found in populations of the three species in the Wuling Mountains, central China (Hunan Province). Lineage B H15, a satellite of H1, was shared by *Q. acutissima* and *Q. variabilis* populations in the Liaodong Peninsula, northeastern China (Liaoning Province), as well as *Q. acutissima* and *Q. chenii* populations in the Tianmu and Huangshan Mountains, southeastern China (Anhui and Zhejiang Provinces). Lineage B H16, another satellite of H1, was only detected in one ‘*Q. acutissima*‐*Q. chenii*’ pair in the Nanling Mountains, southern China (Hunan and Guangdong Provinces).

In these analyses, we also considered high‐frequency haplotypes H2/H7 (lineage A) and H6 (lineage C) because the possibility of introgression‐induced sharing of them cannot be ruled out. Twenty‐six of the 39 population pairs shared these three haplotypes (Figure 4; Table S5). Among those, H2 was shared by one ‘*Q. acutissima*‐*Q. variabilis*’ pair in southwestern China and two ‘*Q. chenii*‐*Q. variabilis*’ pairs in southeastern China; H7 was shared by two *Q. acutissima* and two *Q. variabilis* populations across the Liaodong and Shandong Peninsulas, northern China, as well as by four *Q. acutissima*, three *Q. variabilis*, and two *Q. chenii* populations in the mountainous areas of eastern China (e.g., Mufu, Huangshan, and Tianmu Mountains); H6 was shared by five *Q. acutissima*, five *Q. variabilis*, and one *Q. chenii* population in the mountainous areas of central and southeastern China (e.g., Qinling, Funiu, Tongbai, Dabie, and Tianmu Mountains).

## 4 DISCUSSION

We investigated the cpDNA variation in East Asian Cerris oaks based on the sequence data from 93 wild populations sampled throughout China. We found that the level of interspecific differentiation at cpDNA markers (*F*_CT_ = 0.03; Table 2) is much lower than that reported for nuclear markers (*F*_CT_ ≥0.15; Chen, Zhang, et al., 2021; Li et al., 2022; Liang et al., 2022). These results indicate that cpDNA is less efficient in discriminating the three closely related oaks. The extensive interspecific sharing of cpDNA haplotypes may arise from convergence, past and ongoing introgression/hybridization, and retention of ancestral polymorphism (Acosta & Premoli, 2010; Nevill et al., 2014; Palmé et al., 2004). In our case, convergence seems to be unlikely because identical mutations are low‐probability events and more than 40% of the haplotypes were shared by at least two species. Furthermore, all Fagaceae (and most Fagales) plastomes are notably conserved, that is, have generally low mutation fixation rates (Simeone et al., 2016; Yang, Hu, et al., 2018). The latter two explanations are possible because both randomly and locally distributed haplotypes were interspecifically shared, coinciding with the expectations of shared ancestry and introgression, respectively (Zhou et al., 2017).

### 4.1 Retention of ancestral polymorphism explains the sharing of randomly distributed haplotypes among East Asian Cerris oaks

Our analyses revealed three non‐species‐specific plastid lineages in East Asian Cerris oaks (Figure 1): the most ancestral lineage C is concentrated along the central Chinese mountain ranges; lineage B and its derivate, lineage A are widespread. A similar topology was also observed in the network generated for a single species or a pair of species using more cpDNA markers (see Table S6 for relationships among haplotypes identified in previous work; Chen et al., 2012; Li et al., 2019, 2022; Zhang, Li, et al., 2015). In these studies, the divergence among lineages A, B, and C was supported by more parsimony‐informative sites in the *trnS* ^(GCU)^‐*trnG* ^(UCC)^ and *trnS* ^(GCU)^‐*trnT* ^(GGU)^ regions. These non‐species‐specific lineages hint that the initial differentiation of plastid sequences of East Asian Cerris oaks is independent of the speciation process that formed the modern‐day species; the three modern‐day East Asian Cerris oaks share an ancestral plastid gene pool, which may have split into three lineages before the formation of modern species (Koch & Matschinger, 2007; Premoli et al., 2012; Simeone et al., 2016; Vitelli et al., 2017).

The internal haplotypes of the three major plastid lineages, H2/H7 (lineage A), H1 (lineage B), and H6 (lineage C), presented a relatively high frequency and a wide geographic distribution across populations of the three species (Figure 1), coinciding with the expectation of shared ancestral polymorphism (McGuire et al., 2007). According to the coalescent theory, common internal haplotypes that have more mutational connections and broader geographic distributions are more likely to be ancestral haplotypes (Posada & Crandall, 2001). If all the four haplotypes were present in the common ancestor and had been inherited by the extant taxa, they are expected to be randomly and widely distributed throughout the ranges of the descendant species (McGuire et al., 2007; Zhou et al., 2010; Zhang et al., 2013).

Compared with H1, the two internal haplotypes of the lineage A (H2/H7) are less frequent (Table S4). Previous studies have shown that in the network generated by more cpDNA markers, both H2 and H7 split into several separate haplotypes that were derived from a common missing ancestral haplotype (Table S6; Li et al., 2022). These results indicate that H2 and H7 themselves may only reflect part of the ancestral distribution of the lineage A. Within the H2 lineage, the ancestral type was only found in southwestern China, while the derived type was detected in both northwestern and southeastern China (Li et al., 2022; Zhang, Li, et al., 2015). Such a pattern suggests that the ancestor of the H2 lineage may have migrated from southwestern China to other regions. During this process, the H2 lineage may have diverged among multiple refugia (Zhang, Li, et al., 2015), thus leaving a scattered distribution throughout China. In contrast, the H7 lineage was mainly detected in central and eastern China (Figure 1), of which one member was fixed in all the populations of *Q. variabilis* in Japan (Chen et al., 2012). Such a result implies that the ancestor of the H7 lineage may have diverged locally in central and eastern China and further expanded eastward to Japan probably through the East China Sea land bridge during the glacial periods (Sakaguchi et al., 2012; Wang et al., 2022).

Among the three lineages, the lineage C was most directly linked to the outgroups, specifically the western Eurasian species of sect. *Cerris* and species of closely related oaks of the sister sect. *Ilex* (Figure 1; cf. Simeone et al., 2016: ‘Cerris‐Ilex’ lineage; Zhou et al., 2022: sect. *Cerris*‐sect. *Ilex* p.p. core clade within the Eurasian ‘Old World’ Fagaceae plastid clade). Thus, it represents an ancient plastid lineage of East Asian Cerris oaks. Notably, the haplotypes of this lineage, including the ancestral haplotype H6, were not observed in southwestern China, suggesting that the ancestor of the lineage C, hence, all East Asian Cerris plastomes, originated outside this area. Secondary introgression/hybridization with sym‐ or parapatric sect. *Ilex* oaks (chloroplast capture) as found in the case of Mediterranean oaks (e.g., Simeone et al., 2018; Vitelli et al., 2017) can be ruled out: all haplotypes, including the putatively most ancestral lineage C are restricted to East Asian members of sect. *Cerris*.

Consistent with our results, Zhang et al. (2020) showed that complete chloroplast genome sequences of three *Q. acutissima* trees did not cluster together. Indeed, one of those from northeastern China presented the haplotype H7 (Zhang et al., 2020), while the other two from northwestern and eastern China shared H1 with two *Q. variabilis* and *Q. chenii* individuals (Li et al., 2018; Yang, Hu, et al., 2018). Thus, it is reasonable to see that some *Q. acutissima* trees were grouped with heterospecific trees, rather than conspecific trees, supporting our conclusion that the plastid phylogeny of East Asian Cerris oaks is decoupled from taxonomic boundaries.

### 4.2 Hybridization contributes to the sharing of locally distributed haplotypes among East Asian Cerris oaks

East Asian Cerris oaks share several narrowly distributed and derived haplotypes/haplotype lineages (e.g., H8/H14, H15, H16, and H18) that could point to chloroplast capture, that is, chloroplast of one species being transferred to another through introgression (Figure 4; Acosta & Premoli, 2010; Zhou et al., 2017). The chloroplast capture events have been frequently postulated between closely related oak species in Europe (e.g., Dumolin‐Lapègue et al., 1999; Petit et al., 2004; Simeone et al., 2016; Tekpinar et al., 2021), Northern Africa (e.g., Belahbib et al., 2001), and North America (e.g., Cavender‐Bares et al., 2015; Whittemore & Schaal, 1991; Zhang, Hipp, & Gailing, 2015). Our present study indicates that such phenomena are also common among their congeners in East Asia, where more than 100 oak species occur but only a limited number of reports on the plastid introgression patterns of native oaks are available (e.g., Chen, Zeng, & Zhang, 2021; Li et al., 2022; Lyu et al., 2018; San Jose‐Maldia et al., 2017; Yang, Di, et al., 2016; Zeng et al., 2011).

The locally shared haplotypes were largely concentrated in mountainous areas of central and eastern China (Figure 4). Previous studies have shown that these mountainous areas may have been glacial refugia for many temperate and subtropical tree species (Qiu et al., 2011), including *Q. acutissima* (Zhang, Li, et al., 2015; Zhang et al., 2018), *Q. variabilis* (Chen et al., 2012), and *Q. chenii* (Li et al., 2019). These results suggest that the three oak species may have had multiple isolated but shared glacial refugia, thus allowing long‐term coexistence and historical introgression in different areas (Li et al., 2022; Soliani et al., 2012; Thomson et al., 2015). Indeed, the persistence of East Asian Cerris oaks in these regions may be traced back to an earlier time as evidenced by the rich and widespread fossil records dating to the Miocene and Pliocene (e.g., Shanwang in northern China and Mingguang in southeastern China; Barrón et al., 2017; Momohara, 2016; Song et al., 2000; Zhou, 1993). Such a long history would provide sufficient time for East Asian Cerris oaks to share plastid genomes through introgression. Using nuclear markers, a recent study has shown that a mid‐Pliocene contact zone between the already isolated *Q. acutissima* and *Q. chenii* may have occurred in central and eastern China, where more than 70% of the putative hybrids were concentrated (Li et al., 2022). Furthermore, a relatively high level of introgression was also detected in sympatric populations of *Q. acutissima* and *Q. variabilis* in central and eastern China (Fu et al., 2022). These results support our inference that interspecific sharing of narrowly distributed haplotypes in this region is more likely to be a result of introgression.

A clear case of recent and past introgression from the most widespread *Q. acutissima* into its sister species *Q. variabilis* and their earlier diverged sibling *Q. chenii* (cf. Hipp et al., 2020) is H15. This satellite haplotype of H1 (lineage B) shows a disjunct distribution in central‐eastern and northeastern China. In the Huangshan and Tianmu Mountains of central China (populations CHS/CTM), it is quasi‐private to *Q. chenii*, and only found in a single (out of 19 screened) individual of the *Q. acutissima* population at the Tianmu Mountain, a forest rich in Tertiary relicts (Wang, 1961). The same haplotype is private to the northeasternmost *Q. variabilis* population in our data (Liaodong Peninsula), where it's again shared by one out of 12 individuals of nearby *Q. acutissima* populations. That the same satellite haplotype evolved in a northeastern *Q. variabilis* and two ~1100 km afar southeastern *Q. chenii* populations may be due to fixation of a convergent mutation. However, in the overall conservation of plastid DNA in oaks, it may as well represent a common geographic origin. Once more widespread, the now‐extinct northeastern *Q. chenii* population was introgressed by *Q. variabilis* in northeastern China and its private plastome was captured. *Q. acutissima* captured this potential *Q. chenii* plastome when introgressing at a large scale into its sibling species.

The local sharing of haplotypes may also be influenced by demographic history and the complex landscapes of East Asia. During the Pleistocene, local plants like oaks underwent repeated range contractions and expansions (e.g., Fan et al., 2018; Tian et al., 2015; Ye et al., 2018). These events would increase the level of genetic admixture and promote the spread of some locally distributed haplotypes across multiple refugia (Zhang et al., 2018). Additionally, the geographic ranges of the three species encompass a complex landscape made up of numerous north–south and east–west oriented mountain ranges (Tang et al., 2006). These mountains have not only provided multiple marginal refugia but also offered dispersal corridors for range expansions, thus allowing the migration of individuals with the same haplotypes between different refugia in response to Pleistocene climatic fluctuations (Tian et al., 2018).

Our findings support that local exchange of chloroplast genomes results in an excess of similar haplotypes between nearby populations from different species (Table 3). However, such an effect was not found in the ‘*Q. chenii*‐*Q. variabilis*’ pair, indicating that the level of local introgression differs among species pairs, probably related to the degree of co‐occurrence in the overlapping ranges (Dumolin‐Lapègue et al., 1999), today or in the past. Indeed, we observed only a few cases where *Q. chenii* and *Q. variabilis* coexist in the wild (e.g., the Lushan Mountains, Jiangxi Province) and our dataset did not include any pair of populations that belong to these two species and are separated by less than 1 km. In contrast, mixed stands of *Q. acutissima* and *Q. chenii* or *Q. variabilis* are commonly observed in the field. The interspecific gene identities for sympatric populations of these two species pairs (0.62; Table S7) are comparable with those found in two other pairs of Euro‐Mediterranean oak species, *Q. robur*/*Q. pubescens* (0.67; Dumolin‐Lapègue et al., 1999; Petit, Latouche‐Hallé, et al., 2002) and *Q. suber*/*Q. ilex* (0.57; Belahbib et al., 2001).

## 5 CONCLUSIONS

Our study demonstrates that the haplotype sharing pattern among East Asian Cerris oaks reflects the imprints of both shared ancestral polymorphism and repeated phases of secondary gene flow/reticulation via introgression/hybridization. The three major plastid lineages presented an overlapping distribution, especially in central and eastern China, which differs from that of other Fagales species in Europe and South America by lacking an obvious geographic west–east or north–south structuring. In these species, cpDNA lineages shared among closely related species are partitioned longitudinally or latitudinally, mirroring a history of introgression among multiple isolated refugia (Acosta & Premoli, 2010; Petit, Csaikl, et al., 2002; Premoli et al., 2012). In contrast, East Asia is characterized by complex landscapes and relatively stable climates, which not only allowed the long‐term persistence of ancestral lineages but also connected the survived populations across refugia (Qiu et al., 2011; Tang et al., 2006; Tian et al., 2018). These factors contribute to the overlapping distribution of shared plastid lineages among East Asian Cerris oaks.

## AUTHOR CONTRIBUTIONS

**Yao Li:** Conceptualization (equal); data curation (lead); formal analysis (lead); investigation (equal); writing – original draft (lead). **Lu Wang:** Formal analysis (supporting); investigation (equal); writing – review and editing (equal). **Xingwang Zhang:** Formal analysis (supporting); investigation (equal); writing – review and editing (equal). **Hongzhang Kang:** Resources (equal); writing – review and editing (equal). **Chunjiang Liu:** Resources (equal); writing – review and editing (equal). **Lingfeng Mao:** Supervision (equal); writing – review and editing (equal). **YanMing Fang:** Conceptualization (equal); funding acquisition (lead); supervision (lead); writing – review and editing (equal).

## CONFLICT OF INTEREST

None declared.

## Supporting information


**Appendix S1** Supplementary Figures

**Appendix S2** Supplementary Tables

## Data Availability

Sequence data of East Asian Cerris oaks are available on GenBank (http://www.ncbi.nlm.nih.gov/genbank/) under accession numbers JF753573‐JF753598 (*Q. variabilis*), KT152178‐KT152200 (*Q. acutissima*), MH924168‐MH92419 and OL455916O‐L455917 (*Q. chenii*).
